# Biochemical insight into the prion protein family

**DOI:** 10.3389/fcell.2015.00005

**Published:** 2015-02-11

**Authors:** Danica Ciric, Human Rezaei

**Affiliations:** Virologie et Immunologie Moléculaires, Institut National de la Recherche AgronomiqueJouy-en-Josas, France

**Keywords:** shadoo, doppel, PrP, prion protein, amyloid, evolution, dynamic systems

## Abstract

Prion protein family comprises proteins, which share not only similarity in their primary structure, but also similarity in their fold. These two groups of similarity presume a parceling in their respective biological function through the common biochemical properties. In this review, biochemical and structural similarities of PrP and two other proteins, Doppel and Shadoo, are evocated. Some evidence demonstrating respectively similarity between PrP N-terminal and C-terminal domain with respectively Shadoo and Doppel is presented. We extended primary structure similarity analysis to the other PrP subdomain as 166-176 polyNQ domain and compare it to proteins using aggregation as a support for structural information transference and structural epigenetic. Finally, we questioned if prion protein family have conserved the PrP structural bistability, which should be at the origin of Prion phenomenon and if Prion pathology is not, ultimately, an exaptation of the physiological propensity of PrP to undergo a structural switch and polymerize.

## PRNP and its paralogs

When prion gene was firstly identified it was named “sine,” to indicate scrapie incubation period of the ME7 scrapie agent in mice (Dickinson et al., 1968), later it was shown that this gene corresponds to the murine prion gene, and is linked to the Prn-i gene, which determines incubation time in experimental scrapie (Oesch et al., 1985; Carlson et al., 1986). Human PRN gene locus contains three genes: PRNP, PRND, and a novel gene PRNT (Makrinou et al., 2002). Cellular prion protein (PrP^C^) is encoded by PRNP gene as a single copy. This last has been mapped on the mouse chromosome 2, and on chromosome 20 in the human, where it was mapped to band 20p12-3pte (Sparkes et al., 1986). Human and hamster PRNP consists of two exons, with open reading frame (ORF) located in exon 2. In contrast PRNP of mouse, sheep, and rat contain three exons, with the entire ORF located in exon 3 (Lee et al., 1998).

First prion-related gene, Doppel (Dpl) or “downstream prion protein-like gene,” was discovered during sequencing of cosmid clones, isolated from a Prnp^b/b^ mouse (I/LnJ-4), due to the effort for characterization of the locus around PRNP (Moore et al., 1999). The gene encoding Dpl labeled PRND is located at the same locus with the PRNP gene. The PRND is a single copy gene and is located on the chromosome 2 in mouse 16 kb downstream from PRNP, and at chromosome 20 in human 27 kb downstream, and 52 kb downstream in ovine (Moore et al., 1999). PRND consists of two exons in human, or 4 and 5 exons in mouse depending on the different splicing. The two major transcripts are encoded by the part of exon 3 and exon 4 (Flicek et al., 2014). Current genomic evidence indicates that Dpl was present in the last common ancestor of tetrapods, but was lost in birds since there divergence from reptiles (Harrison et al., 2010). The third member of PRN locus, PRNT gene, was discovered 3 kb downstream from PRND. Even if these three gens are evolutionary related they show low primary structure homology which could suggest distinct biological function.

A new gene outside of PRN locus was discovered by Premzl in 2003 during an exploration for potential homolog of PrP in the in the NCBI non-redundant protein database. The gene coding for Shadoo protein was labeled SPRN and is located at the chromosome 7 in the mouse it has two exons, but the second exon has ORF. Unique transcript of 3374 bps is translated in to the protein product of 147 residues in mouse (Watts and Westaway, 2007). In the Enseml database 16 ortholog sequences of mouse SPRN gene was published in four classes of *Vertebtates* from bony fishes (*Osteichthyes)*, reptiles (*Reptilia*), birds (*Aves*), and mammals (*Mammalia*) (Flicek et al., 2014).

Comparison of predicted amino acid sequences of Sho orthologs showed highly conserved signal peptide responsible for exportation and one Arg-rich repetitive region containing up to six tetra-repeats of consensus XXRG. Moreover, Sho has a hydrophobic region of 20 residues, with strong homology to PrP 106–126 poly Ala segment. Sho's C-terminal domain contains a conserved NXT glycosylation motif and signal peptide predicated for glycophosphotidylinositol (GPI)-anchor attachment (Premzl et al., 2003).

## The evolutionary origin of prion genes

Bioinformatics analyses of PRN loci revealed the evolutionary descent of prion genes from an ancestral ZIP metal ion transporter (Ehsani et al., 2011). During the emergence of metazoa, a cysteine-flanked core domain was inserted, or de novo arose, in a pre-existing ZIP ancestor gene to generate a prion-like ectodomain in a sub-branch of ZIP genes. Approximately a half-billion years later, a genomic insertion of a spliced transcript coding for such a prion-like ZIP ectodomain may have created the prion founder gene (Ehsani et al., 2011). Premzl and colleges were annotated the prion gene family (PrP-GF) in 42 complete eukaryotic genome assemblies, uncovering new genes and pseudo genes. According to this evidence it is likely that the Dpl gene was present in the last common ancestor of *Tetrapoda*, but it was lost in the bird lineage, since its divergence from reptiles. It has been suggested that PRNP and SPRN have evolved from the same ancestral gene into genes that may still share some functions, but may also have also gained new biological roles (Premzl et al., 2004). SPRN gene in mammals and fishes has conserved their genomic position. It is located close to the proximal adjacent gene, encoding a GTP-binding protein (GTP). This gene has tail-to-tail orientation relative to SPRN and it is conserved from fishes to mammals. The other most proximal gene encodes an amine oxidase (AO), is conserved between Fugu (*Arothron sp.)* and mammals, and it has also tail-to-tail orientation with SPRN. The block of three genes, with its conserved gene order (AO–GTP–SPRN) and orientation is an example of conserved contiguity between fishes and mammals, strongly indicates gene orthology. The genes distal to SPRN are not conserved between mouse and human indicating a chromosome rearrangement in either the mouse or human genome (Premzl et al., 2003). The proteins coded by PRNP and his two paralogs PRND and SPRN are grouped in the prion protein family (Prion, Dpl, and Shadoo protein respectively). In contrast, protein product of PRNT gene does not share any distinctive homology with any of proteins of PrP-GF excluding any functional relation (Harrison et al., 2010).

## The PrP protein

**Figure 1:**
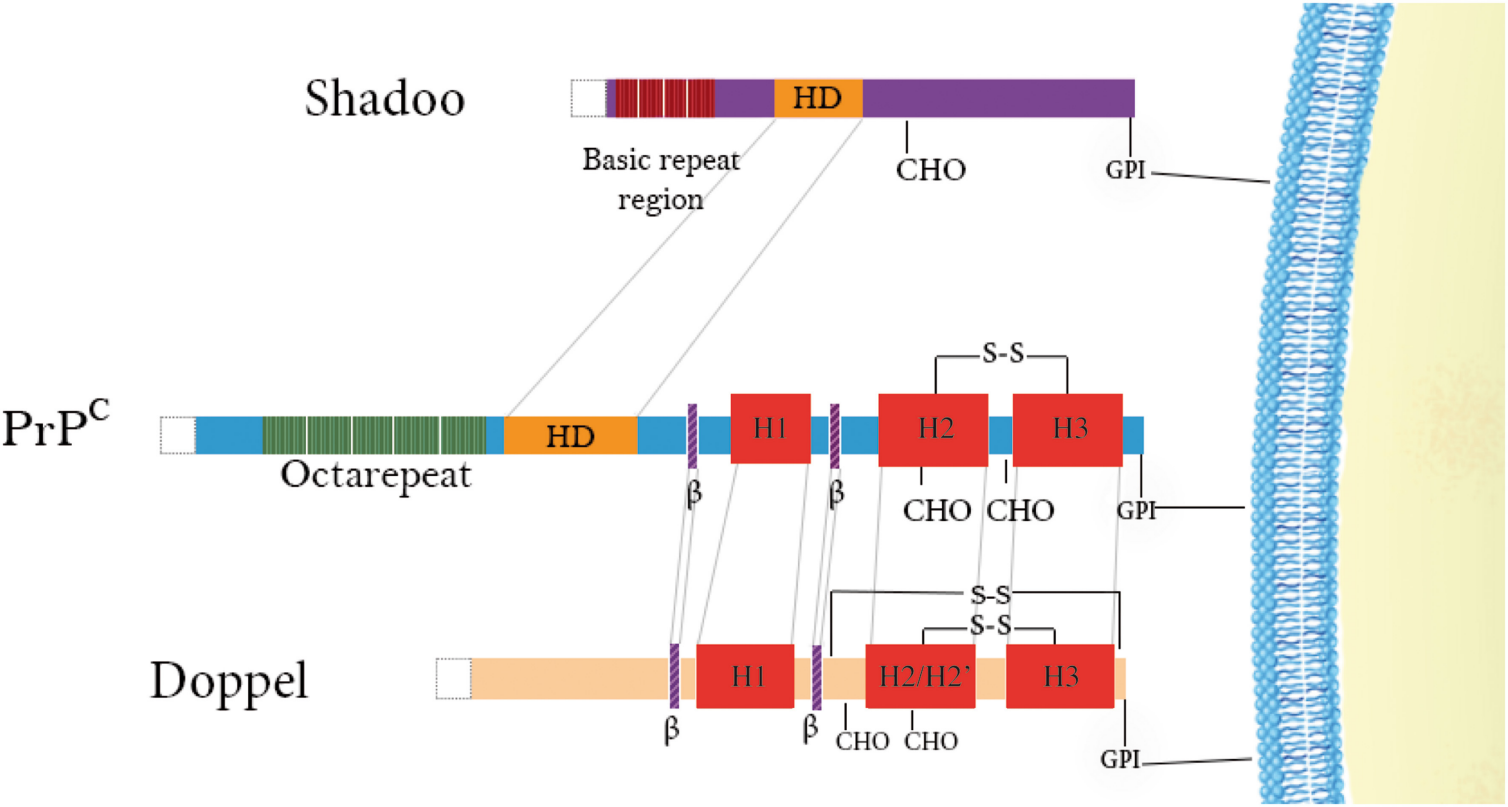
**Schematic representation of the domain architecture of PrP family members**. Doppel and PrP^C^ have structured C-terminal domains consisting of 3 α-helices (in red) and 2 short β-strands (in purple) as well as positively-charged N-terminal regions. Disulfide bridges are indicated above the proteins (–S–S–) and N-glycosylation sites (CHO) are denoted below the proteins. Repetitive regions are found in both PrP^C^ (green) and Shadoo (dark red) with the former possessing octarepeats binding copper and the latter possessing tetrarepeats rich in arginine and glycine residues. A conserved hydrophobic domain is also illustrated in PrP^C^ and Shadoo (yellow) N-terminal signal sequences are cleaved in all of them (white).

When PrP coding sequences were compared in 26 mammalian species it was found that part of sequence for glycosylation sites, positions of cysteines responsible for formation of disulfide bridge, and sequence for putative hydrophobic transmembrane region (Zhang et al., 1997) are perfectly conserved (Van Rheede et al., 2003). Human PrP precursor protein consists of 253 amino acids. It is processed in the ER and Golgi complex during the transport to the cell surface. In the ER its N-terminal signal sequence of 22 residues is cleaved, as well as 23 residues from C-terminal part, after addition of glycosyl phosphatidylinositol (GPI) anchor (Yusa et al., 2012). PrP is properly folded before transporting to the Golgi complex, where it can be differently glycosylated at N181 and N197 position. In cell surface, PrP^C^ can exist in unglycosylated, monoglycosylated, and diglycosylated form (Meyer et al., 1986). Mature PrP^C^ consists of 208 residues (human numbering). It is cell surface glycoprotein, attached to the membrane thought GPI anchor (Figure 1). The N-terminal domain of PrP consists of positively charged amino acid sequences and an octapeptide repeat sequence, which can bind copper ions. Middle region between residues 106–126 constitute conserved hydrophobic domain (HD) rich with alanine and valine. This last segment has been reported to be involved in several regulatory processes (Rezaei-Ghaleh et al., 2011; Béland and Roucou, 2012) and have been reported to be able to span membrane (James et al., 1997).

**Figure 2:**
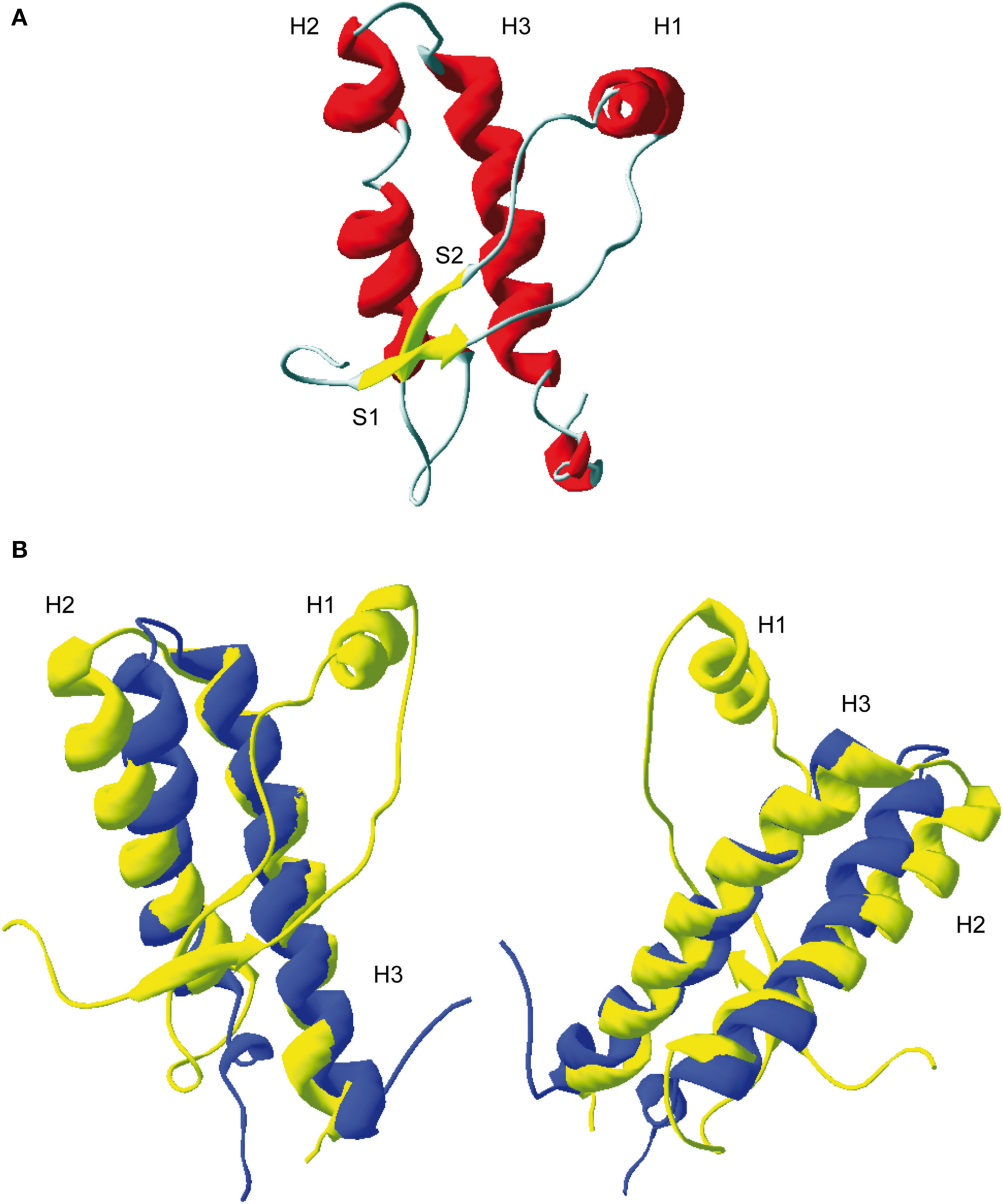
**3D structure of residue 124-231 of recombinant PrP**. **(A)** Ribbon representation of the C-terminal domain of murine PrP (PDB: 1AG2). H1, H2, and H3 represent respectively helix 1, helix 2, and helix 3. S1 and S2, β-sheet 1 and 2. **(B)** Superposition of ovine ARQ structure (in yellow, PDB: ITPX) with the structure of purified ovine H2H3 domain (in blue, residue 169–234, PDB: 2KTM). Left and right represent two different view of the superimposition. As shown, the H2H3 domain adopts quasi-similar fold the full-length globular domain indicating that segment S1-S2-H1 does not contribute the H2H3 folding.

Tertiary structure of PrP globular domain has been resolved by NMR in 1996 (Riek et al., 1996) (Figure 2A). Since this first 3D structure, PrP tertiary structure of several other mammalian species have been resolved (Lysek et al., 2005). All of them, revealed similar and highly conserved fold. Moreover, the comparison between extractive PrP^C^, purified from bovine brain, and bovine recPrP, produced in *E.coli*, revealed similar fold, meaning that glycosylation and GPI anchor did not affect general PrP fold (Hornemann et al., 2004). The PrP 3D structure reveals a globular domain, which contains three α-helices comprising the residues 144-154 (H1), 173-194 (H2), and 200-228 (H3) and a short anti-parallel β-sheet comprising the residues 128–131 (β1) and 161–164 (β2). Within the globular domain there is three loops, between residues 167–171, at the end of H2 residues 187–194, and in the C-terminal part of H3 residues 219–228. PrP have a disulfide bridge between H2 and H3 helixes. The reduction of this S–S bond *in vitro* has been reported to be at origin of structural switch and formation of amyloid fibrils (Jackson et al., 1999). Moreover, it was demonstrated that the H2H3 segment constitutes an independent folding unit (Adrover et al., 2010; Xu et al., 2011) (Figure 2B).

## Doppel protein

**Figure 3:**
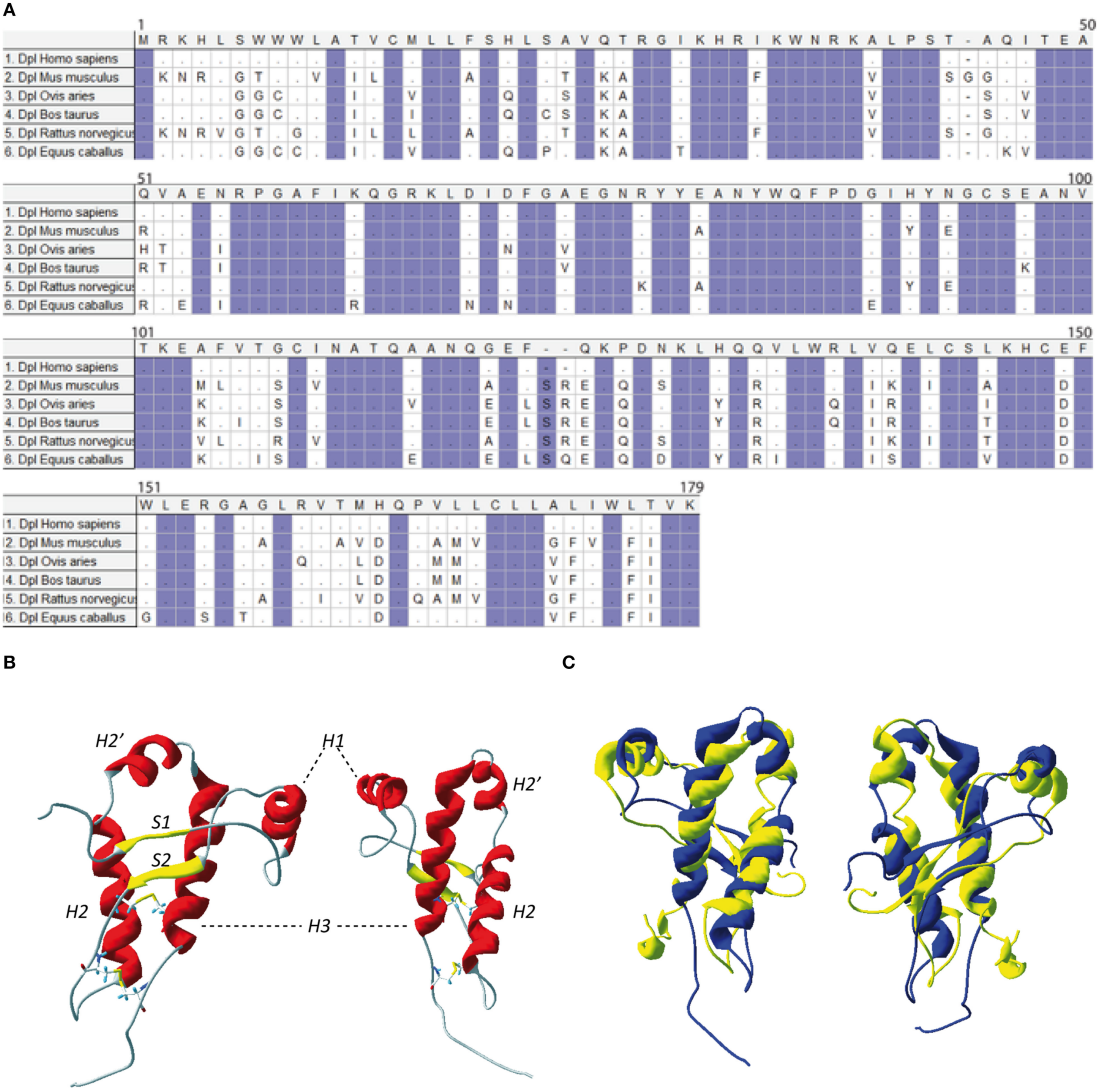
**(A)** Protein sequence of Doppel. Multiple sequence alignment for Doppel protein sequences of different species of vertebrates. Highlighted conserved domains are in violet. Dots refers to identical residues, (-) represents gaps. **(B)** Two different view of mouse Dpl structure (PDB:1I17). **(C)** Two different views of the superimposition of the mouse PrP structure (in yellow, PDB: 1AG2) and mouse Dpl structure (in blue, PDB: 1I17).

PRND gene coding Dpl a 179 residues protein, sharing 25% identity with PrP globular domain. As PrP, Dpl has a cell surface exportation amino acids signal sequence at its N-terminus (1–27 residues) and GPI anchoring signal (from 156 to 179), at its C-terminal domain. Dpl is processed in the ER and Golgi as PrP and has two glycosylation sites, one at the 111 residues in the form N-X-T occurring and second non-conserved N-V-T Asn-linked glycosylation site at residue 99 (Figure 3A). The GPI anchor is predict to be attached at Gly155. The Dpl, as PrP, is attached to outer cell surface trough GPI anchored (Silverman et al., 2000). Despite the fact that both Dpl and PrP^C^ are attached to the rafts, it was reported that they are attached to distinct microenvironments and not in the same raft domains. This observation makes the authors to propose that even though PrP and Dpl share some identities they might have distinct functions (Shaked et al., 2002).

NMR structure of the recombinant human Dpl protein shows a short flexibly disordered N-terminal domain comprising residues 24–51, and a globular domain extending from residues 52–149 (Figure 3B). The globular domain contains four α-helices comprising residues 72–80 (α1), 101–115 (α2a), 117–121 (α2b), and 127–141 (α3), and a short two-stranded anti-parallel β-sheet comprising residues 58–60 (β1) and 88–90 (β2). The C-terminal peptide segment 144–149 folds back onto the loop connecting β2 and α2 (Lührs et al., 2003). Dpl has two disulfide bonds (Cys95–Cys148 and Cys109–Cys143), in contrast to the PrP who has one. This additional disulfide bound could contribute to restriction of Dpl conformational dynamics, making it more rigid. Even if the PrP and Dpl present less than 25% of homology, their folds are similar (Figure 3C).

## Shadoo protein

**Figure 4:**
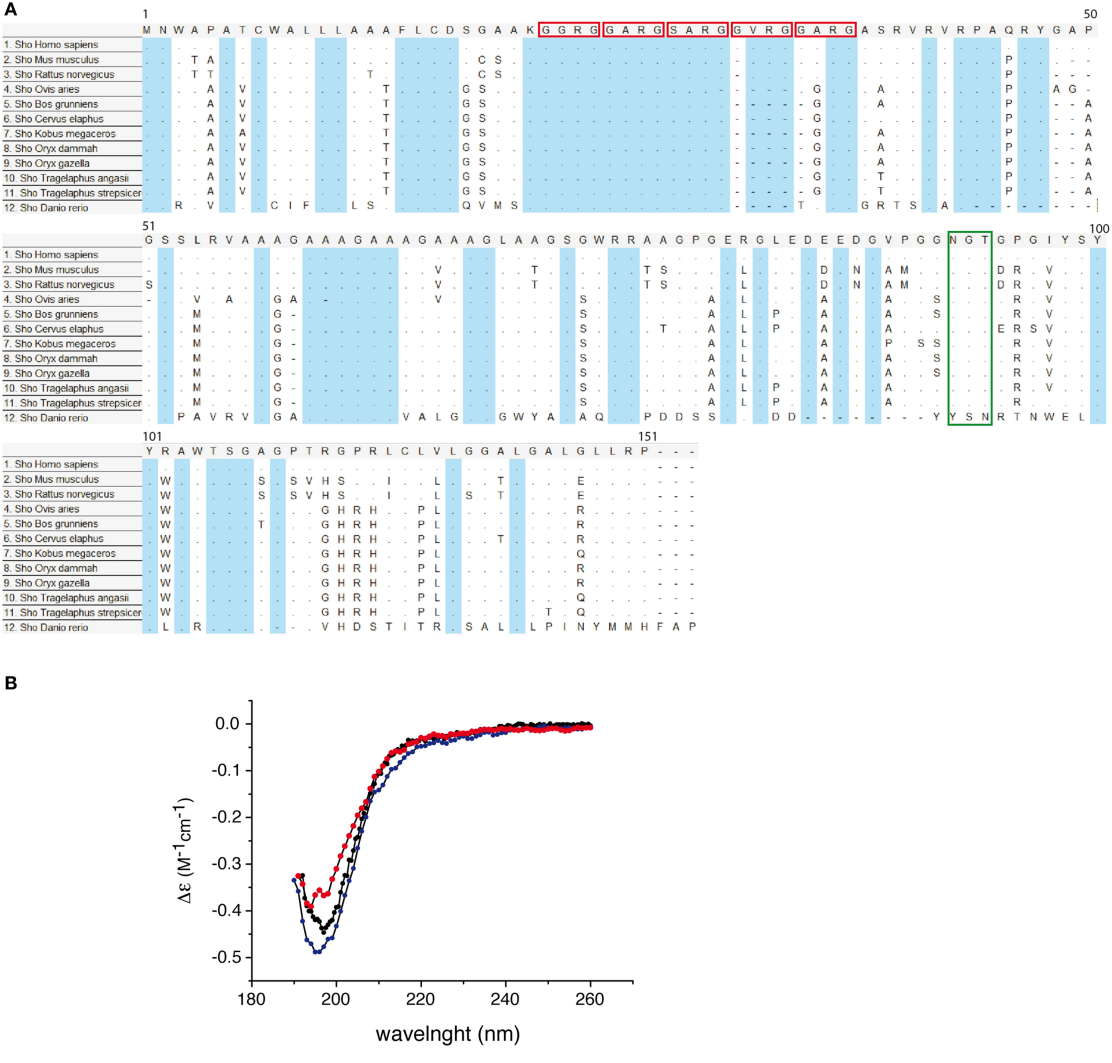
**(A)** Conservation of Sho protein sequences. Alignment of Shadoo protein sequences in 12 species. Multiple alignments of protein referent sequences for Shadoo in vertebrates. In blue color are highlighted conserved domains. Red squares indicate the consensus XXRG repletion. The green box indicates the NXT N-glycosylation signal. The alignment was obtained and color coded by MEGA5 software, dots refers to identical residues, (-) represents gaps, according to MEGA5 convention. **(B)** Circular dichroism (CD) spectra of Sho at pH 3.8, 4.5, and 7.0. CD spectra indicate that Sho adopts a random coil configuration for 3.8 ≤ pH ≤ 7.0.

Nascent protein product encoded by SPRN ORF is subjected to the similar type of processing events as the other members of PrP family. The mature mouse Sho consists of 98 amino acids it is a GPI-anchored protein (Premzl et al., 2003; Watts et al., 2007). Sho also has functional ER targeting signal sequence, and it can be modified with complex glycans and targeted to the outer leaflet of the plasma membrane (Miesbauer et al., 2006). N-terminal segment, from residues 25-42 has very strictly conserved sequence (Figure 4A), with conserved methylation site at Arg27 (GGRGG). In human Sho the RGG repeats are consist of 15 residues: Arg residues are positively charged at physiological pH and 7 Gly residues, 6 of them grouped as dipeptides “GG” giving a high degree of flexibility to the backbone. The increased prevalence of Gly-Gly dipeptides, in the higher Eutherian mammals, could suggest evolutionary pressure for increased flexibility in this domain. In addition, proteins with an RGG-box motif can have RNA-binding function (Thandapani et al., 2013). It is suggested that Sho could bind mRNA directly (Corley and Gready, 2008) and thus play a role in neural plasticity as PrP, through his involvement in neural signaling pathways (Kanaani et al., 2005; Santuccione et al., 2005). HD in the middle of the protein is arranged in five tandem repeats (Figure 4), which consist of GxxxG motifs. These motifs can have a role in organization of transmembrane helixes and packaging of amyloidal fibers (Russ and Engelman, 2000). HD has a high degree of homology with PrP 106–126 domain. The primary structure analysis of Human Sho reveals the existence of a one putative N-glycosilation site (N111). Moreover, the different predication algorithms highly suggest that Sho could be devoid of secondary structure. The CD spectra of recombinant mouse Sho at different pH strongly suggest that Sho adopts a random coil structuration (Figure 4B). These observations strongly suggest that Sho belong to intrinsically disordered proteins (IDP) family.

## Structural dynamic of prion protein family

In early 90th when conventional descriptions of prion pathology failed to describe prion disease, a new theory considering an infectious protein emerged (Griffith, 1967; Prusiner et al., 1982; Prusiner, 1998). The prion theory, now largely extended, stipulate an autonomous structural rearrangement of PrP^C^ into PrP^Sc^ conformer. The prion theory explicitly requires that PrP protein should exist, at least, in two conformations. However, the existence of several strains, for a given PrP primary structure, points out that PrP could exist not only in two conformations, but as plethora of conformations, each associated with a physiopathological state. The fact that PrP could adopt different conformations, in the quasi-similar environments, makes PrP a multi-stable protein, a hallmark of its plasticity. Therefore, one can question why PrP protein, in particular, kept all along the evolution such structural plasticity? The primary structure analysis of prion protein family, could highlight this point. Indeed, divergence of PRNP, PRND, and SPRN conduce to specific differences in PrP, Dpl, and Sho proteins. The existence of additional disulfide bridge in Dpl (Figure 3) highly reduces his plasticity, and therefore reduces Dpl's propensity to adopt several conformations. In terms of PrP-GF evolution, one can consider that additional disulfide bond has been selected to reduce Dpl conformational dynamic and hence, it's self-propensity to undergo a deleterious structural switch. A similar rational could be constructed for Sho. This last protein, during the selection process have discarded segments reported to be involved in the conformational switch of PrP, as it is the case of PrP globular part.

**Figure 5:**
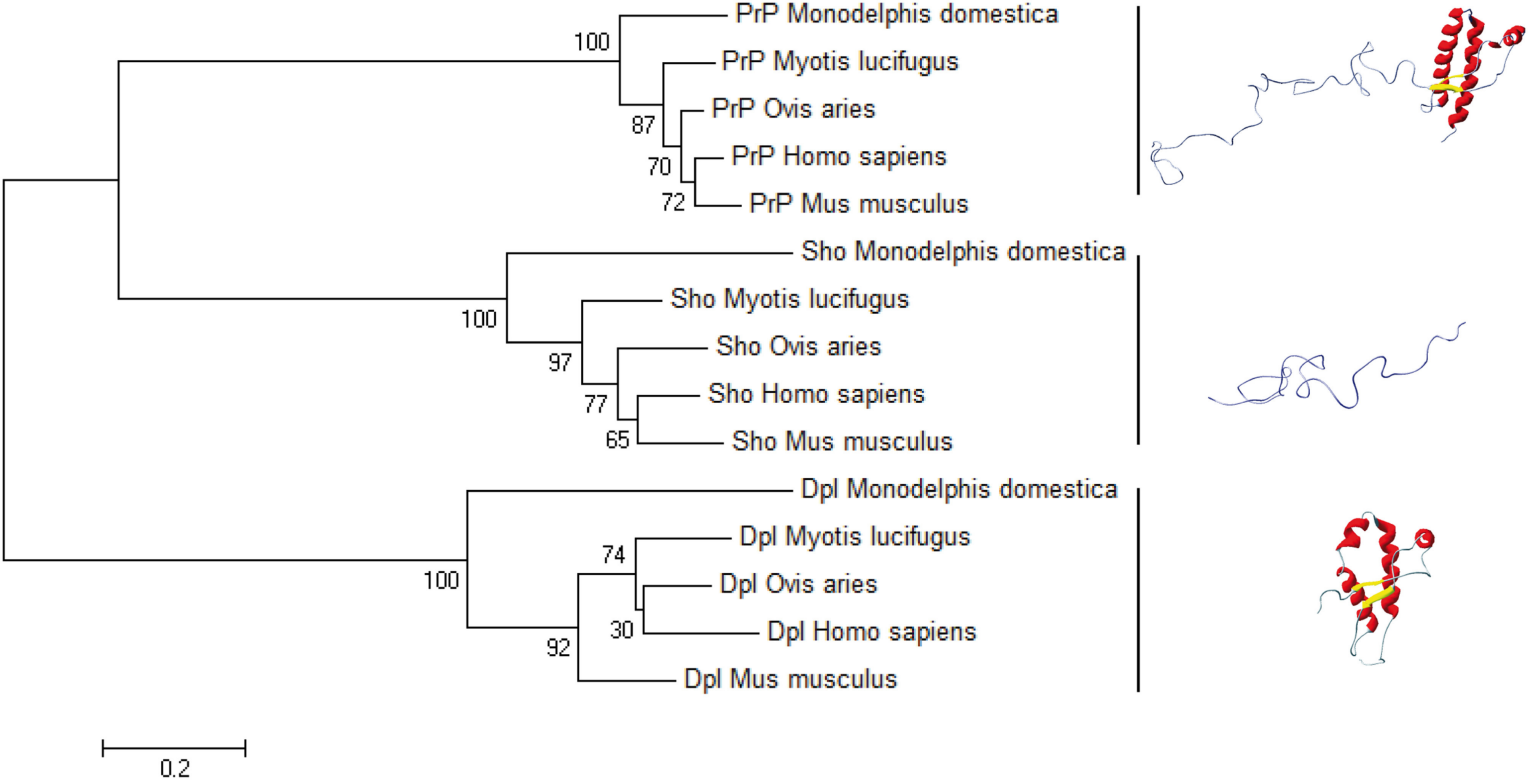
**The bootstrap consensus tree for Prion protein family**. We used MEGA6 to infer the NJ phylogenetic tree in five species of mammals, using the pairwise deletion option for Shadoo, Doppel, and Prion protein sequences retrieved from NCBI Protein database: Mus musculus Dpl (NP_001265449.1), Mus musculus Sho (NP_898970.1), Mus musculus PrP (NP_001265185.1); Ovis aries Dpl (NP_001009261.1), Ovis aries Sho (NP_001156033.1), Ovis aries PrP (NP_001009481.1); Myotis lucifugus Dpl (XP_006096490.1), Myotis lucifugus Sho (XP_006099074.1), Myotis lucifugus PrP (XP_006096489.1); Monodelphis domestica Dpl (CAJ75789.1), Monodelphis domestica Sho (CAJ43800.1), Monodelphis domestica PrP (NP_001035117.1); Homo sapiens Dpl (NP_036541.2), Homo sapiens Sho (NP_001012526.2), Homo sapiens Prp (NP_001073592.1). The horizontal bar indicates 0.2 amino-acid substitution per site.

Another aspect of PRNP gene evolution, in relation with PrP structural landscape, should be also evocated. Compared to Sho and Dpl, the primary structure of PrP is highly conserved among the mammals (Figure 5). This high conservation could be either a hallmark of its folding, linked to its biological function, or linked to the fact that the amount of mutations and variation in the primary structure of PrP could lead to the appearance of deleterious events, as it is the spontaneous conversion. This last phenomenon could be at the origin of PrP mutations responsible of the occurrence of GSS, FFI, and genetic CJD. To better understand this low variability of PrP primary structure we have to consider PrP primary structure in relation with its putative biological function. PrP protein could be segmented into two domains, the N-terminal domain, highly flexible, binding Cu^2+^ ions and the globular domain. So far, most of the PrP biological functions are reported to involve only N-terminal segment. Contribution of the globular domain in the PrP physiological function is not well understood (Béland and Roucou, 2012). The question may arise: why during the mammalian evolution, the propensity of the PrP globular domain to misfold, has not been suppressed, if PrP biological function is only restricted to the N-terminal domain? The answer to this question could be an entanglement between PrP folding and biological function. Indeed, if we consider that PrP biological function is intertwined with its structural bistability in a highly controlled process, evolution should manage with both PrP bistability and the homeostasis of this bistability. One of direct consequences of this hypothesis is that highly controlled PrP conversion could have a physiological role, and that prion pathology could emerge as a breakdown of homeostasis of PrP physiological conversion process, induced by appearance PrP^Sc^.

### Conflict of interest statement

The Associate Editor Jean-Luc Vilotte declares that, despite being affiliated to the same institution as the author Human Rezaei, the review process was handled objectively and no conflict of interest exists. The authors declare that the research was conducted in the absence of any commercial or financial relationships that could be construed as a potential conflict of interest.

## References

[B1] Adrover M. Pauwels K. Prigent S. de Chiara C. Xu Z. Chapuis C.. (2010). Prion fibrillization is mediated by a native structural element that comprises helices H2 and H3. J. Biol. Chem. 285, 21004–21012. 10.1074/jbc.M110.111815 20375014 PMC2898372

[B2] Béland M. Roucou X. (2012). The prion protein unstructured N-terminal region is a broad-spectrum molecular sensor with diverse and contrasting potential functions. J. Neurochem. 120, 853–868. 10.1111/j.1471-4159.2011.07613.x 22145935

[B3] Carlson G. A. Kingsbury D. T. Goodman P. A. Coleman S. Marshall S. T. DeArmond S.. (1986). Linkage of prion protein and scrapie incubation time genes. Cell 46, 503–511. 10.1016/0092-8674(86)90875-5 3015416

[B4] Corley S. M. Gready J. E. (2008). Identification of the RGG box motif in Shadoo: RNA-binding and signaling roles? Bioinform. Biol. Insights 2, 383–400. 19812790 10.4137/bbi.s1075 PMC2735946

[B5] Dickinson A. G. Meikle V. M. H. Fraser H. (1968). Identification of a gene which controls the incubation period of some strains of scrapie agent in mice. J. Comp. Pathol. 78, 293–299. 10.1016/0021-9975(68)90005-4 4970191

[B6] Ehsani S. Tao R. Pocanschi C. L. Ren H. Harrison P. M. Schmitt-Ulms G. (2011). Evidence for retrogene origins of the prion gene family. PLoS ONE 6:e26800. 10.1371/journal.pone.0026800 22046361 PMC3203146

[B7] Flicek P. Amode M. R. Barrell D. Beal K. Billis K. Brent S.. (2014). Ensembl 2014. Nucleic Acids Res. 42, D749–D755. 10.1093/nar/gkt1196 24316576 PMC3964975

[B8] Griffith J. S. (1967). Self-replication and scrapie. Nature 215, 1043–1044. 10.1038/2151043a0 4964084

[B9] Harrison P. M. Khachane A. Kumar M. (2010). Genomic assessment of the evolution of the prion protein gene family in vertebrates. Genomics 95, 268–277. 10.1016/j.ygeno.2010.02.008 20206252

[B10] Hornemann S. Schorn C. Wüthrich K. (2004). NMR structure of the bovine prion protein isolated from healthy calf brains. EMBO Rep. 5, 1159–1164. 10.1038/sj.embor.7400297 15568016 PMC1299192

[B11] Jackson G. S. Hosszu L. L. Power A. Hill A. F. Kenney J. Saibil H.. (1999). Reversible conversion of monomeric human prion protein between native and fibrilogenic conformations. Science 283, 1935–1937. 10.1126/science.283.5409.1935 10082469

[B12] James T. L. Liu H. Ulyanov N. B. Farr-Jones S. Zhang H. Donne D. G.. (1997). Solution structure of a 142-residue recombinant prion protein corresponding to the infectious fragment of the scrapie isoform. Proc. Natl. Acad. Sci. 94, 10086–10091. 10.1073/pnas.94.19.10086 9294167 PMC23313

[B13] Kanaani J. Prusiner S. B. Diacovo J. Baekkeskov S. Legname G. (2005). Recombinant prion protein induces rapid polarization and development of synapses in embryonic rat hippocampal neurons *in vitro*. J. Neurochem. 95, 1373–1386. 10.1111/j.1471-4159.2005.03469.x 16313516

[B14] Lee I. Y. Westaway D. Smit A. F. A. Wang K. Seto J. Chen L.. (1998). Complete genomic sequence and analysis of the prion protein gene region from three mammalian species. Genome Res. 8, 1022–1037. 9799790 10.1101/gr.8.10.1022

[B15] Lührs T. Riek R. Güntert P. Wüthrich K. (2003). NMR structure of the human doppel protein. J. Mol. Biol. 326, 1549–1557. 10.1016/S0022-2836(02)01471-7 12595265

[B16] Lysek D. A. Schorn C. Nivon L. G. Esteve-Moya V. Christen B. Calzolai L.. (2005). Prion protein NMR structures of cats, dogs, pigs, and sheep. Proc. Natl. Acad. Sci. U.S.A. 102, 640–645. 10.1073/pnas.0408937102 15647367 PMC545531

[B17] Makrinou E. Collinge J. Antoniou M. (2002). Genomic characterization of the human prion protein (PrP) gene locus. Mamm. Genome 13, 696–703. 10.1007/s00335-002-3043-0 12514748

[B18] Meyer R. K. McKinley M. P. Bowman K. A. Braunfeld M. B. Barry R. A. Prusiner S. B. (1986). Separation and properties of cellular and scrapie prion proteins. Proc. Natl. Acad. Sci. U.S.A. 83, 2310–2314. 10.1073/pnas.83.8.2310 3085093 PMC323286

[B19] Miesbauer M. Bamme T. Riemer C. Oidtmann B. Winklhofer K. F. Baier M.. (2006). Prion protein-related proteins from zebrafish are complex glycosylated and contain a glycosylphosphatidylinositol anchor. Biochem. Biophys. Res. Commun. 341, 218–224. 10.1016/j.bbrc.2005.12.168 16414019

[B20] Moore R. C. Lee I. Y. Silverman G. L. Harrison P. M. Strome R. Heinrich C.. (1999). Ataxia in prion protein (PrP)-deficient mice is associated with upregulation of the novel PrP-like protein doppel. J. Mol. Biol. 292, 797–817. 10.1006/jmbi.1999.3108 10525406

[B21] Oesch B. Westaway D. Wälchli M. McKinley M. P. Kent S. B. Aebersold R.. (1985). A cellular gene encodes scrapie PrP 27-30 protein. Cell 40, 735–746. 10.1016/0092-8674(85)90333-2 2859120

[B22] Premzl M. Gready J. E. Jermiin L. S. Simonic T. Graves J. A. M. Marshall Graves J. A. (2004). Evolution of vertebrate genes related to prion and Shadoo proteins–clues from comparative genomic analysis. Mol. Biol. Evol. 21, 2210–2231. 10.1093/molbev/msh245 15342797

[B23] Premzl M. Sangiorgio L. Strumbo B. Marshall Graves J. A. Simonic T. Gready J. E. (2003). Shadoo, a new protein highly conserved from fish to mammals and with similarity to prion protein. Gene 314, 89–102. 10.1016/S0378-1119(03)00707-8 14527721

[B24] Prusiner S. B. (1998). Nobel lecture: prions. Proc. Natl. Acad. Sci. U.S.A. 95, 13363–13383. 10.1073/pnas.95.23.13363 9811807 PMC33918

[B25] Prusiner S. B. Bolton D. C. Groth D. F. Bowman K. A. Cochran S. P. McKinley M. P. (1982). Further purification and characterization of scrapie prions. Biochemistry 21, 6942–6950. 10.1021/bi00269a050 6818988

[B26] Rezaei-Ghaleh N. Andreetto E. Yan L. M. Kapurniotu A. Zweckstetter M. (2011). Interaction between amyloid beta peptide and an aggregation blocker peptide mimicking islet amyloid polypeptide. PLoS ONE 6: e20289. 10.1371/journal.pone.0020289 21633500 PMC3102090

[B27] Riek R. Hornemann S. Wider G. Billeter M. Glockshuber R. Wüthrich K. (1996). NMR structure of the mouse prion protein domain PrP(121-231). Nature 382, 180–182. 10.1038/382180a0 8700211

[B28] Russ W. P. Engelman D. M. (2000). The GxxxG motif: a framework for transmembrane helix-helix association. J. Mol. Biol. 296, 911–919. 10.1006/jmbi.1999.3489 10677291

[B29] Santuccione A. Sytnyk V. Leshchyns'ka I. Schachner M. (2005). Prion protein recruits its neuronal receptor NCAM to lipid rafts to activate p59fyn and to enhance neurite outgrowth. J. Cell Biol. 169, 341–354. 10.1083/jcb.200409127 15851519 PMC2171870

[B30] Shaked Y. Hijazi N. Gabizon R. (2002). Doppel and PrP(C) do not share the same membrane microenvironment. FEBS Lett. 530, 85–88. 10.1016/S0014-5793(02)03430-0 12387871

[B31] Silverman G. L. Qin K. Moore R. C. Yang Y. Mastrangelo P. Tremblay P.. (2000). Doppel is an N-glycosylated, glycosylphosphatidylinositol-anchored protein. Expression in testis and ectopic production in the brains of Prnp(0/0) mice predisposed to Purkinje cell loss. J. Biol. Chem. 275, 26834–26841. 10.1074/jbc.M003888200 10842180

[B32] Sparkes R. S. Simon M. Cohn V. H. Fournier R. E. Lem J. Klisak I.. (1986). Assignment of the human and mouse prion protein genes to homologous chromosomes. Proc. Natl. Acad. Sci. 83, 7358–7362. 10.1073/pnas.83.19.7358 3094007 PMC386716

[B33] Thandapani P. O'Connor T. R. Bailey T. L. Richard S. (2013). Defining the RGG/RG motif. Mol. Cell 50, 613–623. 10.1016/j.molcel.2013.05.021 23746349

[B34] Van Rheede T. Smolenaars M. M. W. Madsen O. de Jong W. W. (2003). Molecular evolution of the mammalian prion protein. Mol. Biol. Evol. 20, 111–121. 10.1093/molbev/msg014 12519913

[B35] Watts J. C. Drisaldi B. Ng V. Yang J. Strome B. Horne P.. (2007). The CNS glycoprotein Shadoo has PrP(C)-like protective properties and displays reduced levels in prion infections. EMBO J. 26, 4038–4050. 10.1038/sj.emboj.7601830 17703189 PMC1950727

[B36] Watts J. C. Westaway D. (2007). The prion protein family: diversity, rivalry, and dysfunction. Biochim. Biophys. Acta 1772, 654–672. 10.1016/j.bbadis.2007.05.001 17562432

[B37] Xu Z. Prigent S. Deslys J.-P. Rezaei H. (2011). Dual conformation of H2H3 domain of prion protein in mammalian cells. J. Biol. Chem. 286, 40060–40068. 10.1074/jbc.M111.275255 21911495 PMC3220554

[B38] Yusa S. Oliveira-Martins J. B. Sugita-Konishi Y. Kikuchi Y. (2012). Cellular prion protein: from physiology to pathology. Viruses 4, 3109–3131. 10.3390/v4113109 23202518 PMC3509686

[B39] Zhang H. Stockel J. Mehlhorn I. Groth D. Baldwin M. A. Prusiner S. B.. (1997). Physical studies of conformational plasticity in a recombinant prion protein. Biochemistry 36, 3543–3553. 10.1021/bi961965r 9132005

